# Hydronephrosis in Infants and Children: Natural History and Risk Factors for Persistence in Children Followed by a Medical Service

**DOI:** 10.4137/cmped.s3584

**Published:** 2009-12-16

**Authors:** Kristy VanDervoort, Stephanie Lasky, Christine Sethna, Rachel Frank, Suzanne Vento, Jeanne Choi-Rosen, Beatrice Goilav, Howard Trachtman

**Affiliations:** Departments of Pediatrics and Radiology, Schneider Children’s Hospital of the North Shore-LIJ Health System, New Hyde Park, NY. Email: trachtma@lij.edu

**Keywords:** ureteropelvic junction obstruction, hydronephrosis, renal ultrasound, long-term follow-up

## Abstract

**Background:**

Infants with neonatal hydronephrosis and a normal voiding cystourethrogram (VCUG) are presumed to have ureteropelvic junction obstruction (UPJO). There is little current information about the natural history of children with hydronephrosis or clinical factors that predict resolution of the radiological abnormality.

**Objective:**

To determine the time course until spontaneous resolution of neonatal hydronephrosis and define risk factors for persistence of the abnormality.

**Methods:**

This retrospective single center review examined infants and children <5 years of age with hydronephrosis who were followed for at least 12 months.

**Results:**

136 children were identified (96 male:40 female). The mean age at diagnosis of hydronephrosis was 3.3 ± 9.7 months and 76% of the patients were diagnosed at birth. The hydronephrosis was unilateral in 98 (72%) of cases, and hydronephrosis was at least moderate in severity in 22% of affected kidneys. At last follow-up at 30 ± 10 months, the abnormality had resolved in 77 out of 115 (67%) available patients, 30 (26%) had been referred to urology, and 12 (10%) had persistent hydronephrosis. Severity of hydronephrosis was the only clinical feature that predicted persistence of the abnormality (P < 0.001). There was an association between detection at birth and lack of resolution of hydronephrosis.

**Conclusions:**

Children with hydronephrosis and presumed UPJO and normal kidney parenchyma can be followed for at least 2 years to allow for spontaneous resolution before referral to urology. Serial sonography can be performed at 6 month intervals in uncomplicated cases. More severe hydronephrosis and presence of the lesion at birth may predict infants and children requiring closer observation and referral for possible surgical correction of the hydronephrosis.

## Introduction

Infants with neonatal hydronephrosis can have a range of abnormalities including ureteropelvic junction obstruction (UPJO), ureterovesical junction obstruction, megacystis megaureter, or vesicoureteral reflux (VUR). The second and third entities are fairly uncommon and, therefore, pediatric patients who have hydronephrosis and a normal voiding cystourethrogram (VCUG) are presumed to have UPJO. This abnormality occurs in approximately 1 in every 2,000 live births and accounts for approximately half of the cases of prenatal hydronephrosis.^1^,^2^

In most cases, neonatal hydronephrosis and presumed UPJO gradually resolves without surgical intervention. There is a strong correlation between the Society for Fetal Urology (SFU) grade of hydronephrosis and the likelihood of spontaneous resolution: Grade I resolves in approximately 50% of patients, and grades II, III, IV hydronephrosis resolve in 36%, 16%, and 3% of cases, respectively.^2^ However, there is little information about the natural history of hydronephrosis and the time to resolution of the lesion in current practice when most women undergo serial sonography and screening for renal anomalies during pregnancy. Moreover, there are few guidelines regarding frequency of follow-up visits and referral to urology for more extensive testing such as performance of renal radionuclide scans and possible surgery in children with hydronephrosis who are cared for by general pediatricians and pediatric nephrologists. Additionally, the value of clinical markers such as birth history, urinary tract infection (UTI), and severity of the hydronephrosis as guides in predicting which patients are likely to require urological evaluation has not been adequately studied. Therefore, this review was conducted to determine the time course until spontaneous resolution of neonatal hydronephrosis and presumed UPJO and to identify risk factors for persistent hydronephrosis in a group of children followed by a pediatric nephrology service. The primary target audience of this review is general pediatricians in order to help guide strategies for serial observation and referral to pediatric urology.

## Patients and Methods

### Patients

This retrospective single-site chart review was conducted in all pediatric patients ages 5 and under who were diagnosed with hydronephrosis at our institution between 2001 and 2007 and followed by the Division of Nephrology. The clinical data were collected on pre-approved data collection sheets, the information was deidentified, and the sheet linking the study number with the individual patient was stored in a secure location. The project was reviewed and approved by the Institutional Review Board.

A database maintained at the Division of Nephrology, which includes patient gender, age, and chief complaint was scanned to identify patients with “UPJ obstruction,” “hydronephrosis,” or “obstructive uropathy.” The review of charts with these three diagnostic terms was done to ensure complete ascertainment of patients with hydronephrosis and presumed UPJO. The patients’ medical records were retrieved from active files or off-site storage facilities. Children were included in this review if they had: (1) hydronephrosis without hydroureter detected in a postnatal ultrasound and a negative VCUG; or (2) hydronephrosis without hydroureter in the absence of a negative VCUG if the child had not had a UTI and the parents refused the VCUG. The standard recommendation at this institution is to perform a VCUG in any newborn or infant under 3 months of age with confirmed hydronephrosis. In addition, patients had to be followed for a minimum of 12 months to be eligible for inclusion in the study.

The following data were recorded for patients: date of birth, gender, presence of prenatal hydronephrosis, gestational age/birth weight, history of perinatal complications or UTI, severity of hydronephrosis, age at diagnosis, unilateral or bilateral disease, serial ultrasounds, and outcome, namely resolution of hydronephrosis, persistent hydronephrosis, or referral to urology. The time from diagnosis to each outcome was then calculated by month.

Babies were labeled premature if they were born before 37 weeks of gestation or if their birth weight was less than 2500 grams. Perinatal complications included maternal complications such as gestational diabetes and preeclampsia, congenital abnormalities, and postnatal complications such as respiratory distress syndrome or sepsis/infection. Severity of hydronephrosis of each renal unit was recorded based on the radiology report as mild, mild to moderate, moderate, moderate to severe, or severe. Outcome was recorded as improving, worsening, stable, referred to urology, or resolved.

### Radiology methods

For renal ultrasound studies that were performed at Schneider Children’s Hospital, the procedure was done with a US Transducer using the HDI 5000 or GE Logiq 9 system. The longitudinal and transverse dimensions of each kidney were recorded. Renal ultrasounds were then graded based on anteroposterior mid-renal pelvic diameter (APD) width into the following categories: mild, mild/moderate, moderate, moderate/severe, and severe hydronephrosis. There were no set guidelines for these grades and the radiologists did not use the SFU grading scheme on a consistent basis. The written description of the degree of hydronephrosis was recorded for all studies done at other institutions. For analysis of outcome based on severity of the hydronephrosis, the abnormality was categorized as less than or greater than or equal to moderate.

To validate the description of the grade of hydronephrosis recorded in the radiology reports, a radiologist (JCR) reassessed a random sample of ultrasound images and compared the grade against the interpretations in the original report. Hydronephrosis was graded by directly measuring the APD. In order to classify grades using APD measurements, a general baseline was used, in which normal was 0–4 mm, mild 5–9 mm, moderate 10–15 mm, and severe greater than 15 mm.^3^ Although the radiologist noted the SFU grade during the reassessment of the renal ultrasound findings, these data are not shown because the absence of this method of grading hydronephrosis during the imaging study.

### Statistical methods

Data are presented as mean ± SD. Continuous data and differences in proportion for categorical data were analyzed statistically by the student t-test and Chi-Square test, respectively. Simple logistic regression was performed to determine which clinical and radiological features predicted the outcome (persistence *versus* resolution) of the hydronephrosis at the last follow-up visit. A P value less than 0.05 was considered significant.

## Results

A total of 368 records were identified in the divisional database of which 295 charts were retrieved (80%). Hydronephrosis was diagnosed in 156 children, and 136 of these patients were followed for at least 12 months. This latter group comprised the study cohort and the key clinical features are summarized in Table 1. Fifteen of 117 (13%) children with adequate birth records were premature and 53 of 130 (41%) cases with complete documentation had perinatal complications. Twenty-three children had a UTI, and only 2 of these patients were diagnosed with hydronephrosis at birth. Of the 115 children who were followed to 30 months, 87 (76%) were diagnosed by ultrasound within the first week after birth, while 28 (24%) were diagnosed later in life. Among these patients, 112 (82%) had a VCUG performed within the first 3 years of life and all were normal. The 24 patients with hydronephrosis who did not have a VCUG were similar in all respects to the 112 children who had a negative VCUG, except for the complete absence of UTIs in the former group, making vesicoureteral reflux an unlikely explanation for the hydronephrosis. Seventeen patients were lost to follow-up after 1 year, but included in the study until the time that they were lost. The hydronephrosis was unilateral in 98 (72%) cases resulting in a total of 174 renal units with hydronephrosis. Initial ultrasound diagnosis revealed 135 renal units with hydronephrosis of mild or mild-to-moderate severity, while the hydronephrosis in 39 renal units was moderate or greater in severity (Table 2).

**Figure 1:**
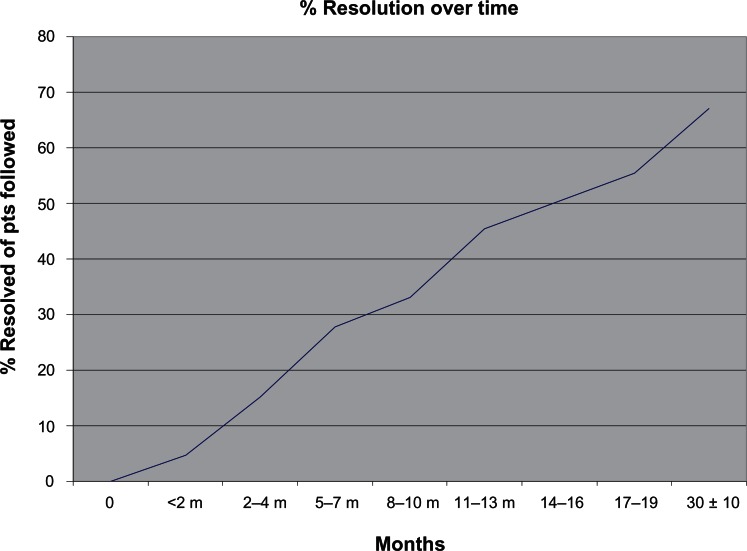
The graph illustrates the percentage of patients available for follow-up at each time point who had resolution of the hydronephrosis and UPJO.

At 6 months 35/126 (28%), at 1 year 55/121 (46%), and at 18 months, 66/119 (56%) children had spontaneous resolution of hydronephrosis. At the last follow-up visit, which took place 30 ± 10 months after the initial diagnosis, 77 out of 115 patients (67%) who were available for assessment had resolution of their hydronephrosis, 30 (26%) had been referred to urology, and 12 (10%) had persistent hydronephrosis (Fig. 1). Among the 77 children whose hydronephrosis ultimately resolved, 8 (10%) had transient worsening at some point during follow-up.

Of the 94 (69%) children diagnosed with mild or mild-to-moderate hydronephrosis, 67 resolved while 15 were lost to follow-up. In the moderate-to-severe cohort, only 10 out of 36 had resolution (Table 3). Among the clinical and laboratory features analyzed, only the severity of the hydronephrosis and detection of the hydronephrosis at birth were associated with persistence of the finding. Prematurity, UTI, and presence of perinatal complications had no significant impact on the likelihood of resolution (Table 4). Simple logistic regression confirmed these findings. Thus, degree of hydronephrosis predicted failure of resolution of hydronephrosis (P < 0.001). In addition, there was a trend for presence of hydronephrosis at birth to predict persistence of the abnormality (P < 0.06) (Table 5).

**Figure 2:**
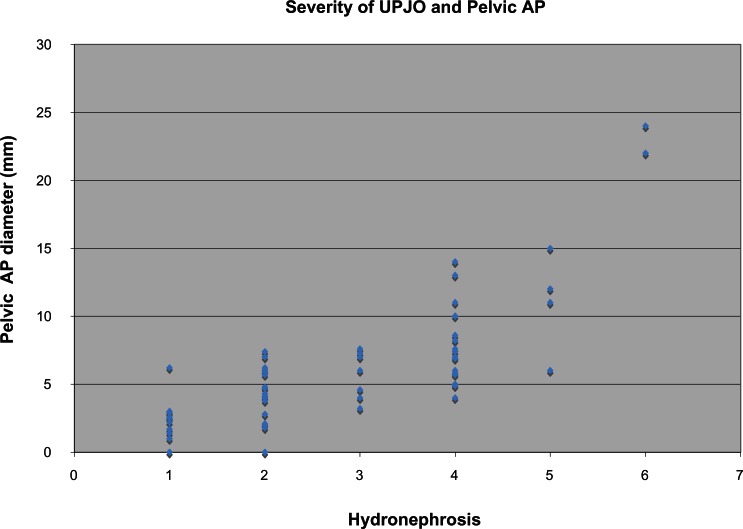
The histogram illustrates the relationship between the anteroposterior pelvic diameter and the grade of hydronephrosis in the 28 patients whose renal ultrasound was reviewed by a second by the pediatric radiologist and who was blinded to the original interpretation. The scale on the abscissa is as follows: 1, resolved; 2, mild; 3, mild-to-moderate; 4, moderate; 5, moderate-to-severe; 6, severe.

Of the 136 patients that were identified in our study, renal ultrasounds of 28 (21%) of the patients were reviewed again to assess the concordance between the written report and the measured degree of hydronephrosis. As illustrated in Figure 2, there was a significant correlation between the severity of hydronephrosis described in the radiological reports and the APD dimensions of the hydronephrosis. In addition, there was close concordance between the verbal and SFU grading of hydronephrosis assigned by the radiologist. There was no overlap between the descriptive categories utilized in this report.

## Discussion

Extensive use of prenatal ultrasound has led to an increased rate of diagnosis of neonatal hydronephrosis, of which UPJO is the most frequent cause.^4^ The postnatal imaging investigations and management of children with hydronephrosis and presumed UPJO is debated due to the high number of cases that resolve without intervention. This retrospective single-center chart review indicates that almost three quarters of cases of hydronephrosis detected in the neonatal period or early childhood resolved spontaneously over 30 months. While 10% of these patients had transient worsening at some time point, they all ultimately resolved. We suspect that some patients referred to urology also have spontaneous resolution of hydronephrosis without intervention. This would underestimate the rate of resolution of hydronephrosis and likely lend further support to our conservative recommendations. The initial severity of the lesion at the time of diagnosis and presence of the hydronephrosis at birth were the only two factors that predicted failure to resolve. The recent time period (2001–2007) of our study reflects the inclusion of children who had prenatal sonograms and reflects the outcomes of hydronephrosis under current standards of antenatal care when the majority of women receive prenatal sonograms regularly during pregnancy.

To our knowledge, this study represents the largest cohort of patients with hydronephrosis followed exclusively by a medical service. In children with hydronephrosis, the question of timing of follow-up imaging studies and their management has significant implications for routine pediatric health care. This study provides support for the practice of monitoring children with hydronephrosis for a longer period of time without mandatory early intervention or referral to urology. Twenty-three percent of neonates followed for 6 years in one study^5^ and 7% of neonates with unilateral hydronephrosis followed for 5 years in another ultimately required surgical intervention.^6^,^7^ In our cohort, only 28% resolved at 6 months versus 67% at 30 months. Twenty-two of the 77 children with resolved hydronephrosis did so after 12 months, supporting the notion that they do not need to be referred to urology for persistent hydronephrosis at 1 year. Furthermore, a few of the children that ultimately resolved had transient worsening, which should alleviate concerns that children with hydronephrosis that worsens need urgent referral and surgical evaluation.

In children with posterior urethral valves, it is suggested that ultrasound imaging be done every 4 months for the first year of life.^8^ However, these patients are more complicated and may require more intensive monitoring of kidney and bladder structure. Our data support the need for less frequent ultrasound examinations in children with hydronephrosis secondary to presumed UPJO. We suggest that in the absence of severe disease or renal parenchyma thinning, the physician can choose to follow the children with serial sonography every 6–12 months for the first 2–3 years of life and observe for improvement. Most children who do require pyeloplasty usually do so within the first 2 years of life.^9^

It is important to acknowledge certain features of our study cohort. We have characterized the patients as having hydronephrosis due to presumed UPJO. The later entity requires urodynamic confirmation with nuclear medicine studies. Thus, a ^99m^Tc-Mag3 renal radionuclide scan with furosemide washout is often part of the assessment of children with moderate-to-severe hydronephrosis.^10^,^11^ In addition, a VCUG should be done to exclude VUR. In our sample of 136 patients, many did not have a radionuclide scan and a VCUG was not done in 24 cases. Nonetheless, based on their clinical features, we suggest that it is justified to describe them as having hydronephrosis with presumed UPJO. This reflects current practice in which parents often refuse a VCUG and the nature of patients followed for the abnormality. The categorization is supported by the generally favorable outcome in which there was resolution of the hydronephrosis in the majority of patients.

Ultrasound examination allows staging of the severity of hydronephrosis in UPJO based on the SFU grades I–IV according to the APD and the appearance of the renal calyces. We described the hydronephrosis in words rather than by the APD and SFU grade because this is likely to be the standard practice in many hospitals. However, we do not consider this a shortcoming of our study, based on the correlation in verbal grade and directly measured APD.

A large meta-analysis by Sidhu et al. showed that Grade I–II hydronephrosis was about 5 times more likely to resolve as compared to higher severity lesions.^12^ Similarly, in our study, hydronephrosis that was mild to mild-to-moderate in severity resolved in 71% of patients, compared to 28% of children with moderate to severe hydronephrosis. This supports the claim that severity of hydronephrosis predicts failure of the lesion to resolve. However, Onen and colleagues found that even with more severe lesions, two-thirds of patients still do not require surgery.^13^ While the correlation between severity and resolution has been previously shown, no other studies have correlated the risk of hydronephrosis at birth and persistence of the lesion. This distinctive finding in our series may allow physicians to use their clinical judgment for extended observation before referral to urology when caring for children with hydronephrosis diagnosed later in life, because they may have an increased chance of resolution.

We acknowledge that there are limitations in our study regarding the natural history of hydronephrosis. This was a single-center, retrospective review and the clinical information surveyed was often unavailable in the medical records. In our study, children were referred both immediately after birth and later in life for evaluation of their hydronephrosis. This could potentially influence our results because the follow-up time period may be different physiologically in terms of kidney development from the time of birth compared to that same interval later in life. The children with hydronephrosis in this study were considered to have presumed UPJO even though a radionuclide scan was usually not done to confirm a urodynamically significant obstruction. Nonetheless, we think our categorization is appropriate and reflects the generally mild nature of this genitourinary tract anomaly. We attempted to ensure that there was consistency in the radiological interpretations of the ultrasound reports. The findings in the sample of ultrasounds that were reviewed by the radiologist indicate that the terminology used in the report is accurate and a reliable marker to guide management. However, physicians should take into account small changes in ultrasound interpretations by different radiologists in regard to “stable” hydronephrosis. Most cases of resolution were reported as no evidence of hydronephrosis by ultrasound. There were a few cases in which the child was clinically well and whose UPJO was considered “resolved.” In these cases, previously mild or mild-to-moderate hydronephrosis was greatly improved but repeat imaging revealed minimal, stable dilatation of the pelvis. Like most areas of medicine, clinical judgment is an important determining factor in specific management decisions.

Our review of hydronephrosis in infants and children is directed at general pediatricians and pediatric nephrologists who are responsible for weighing the choice of continued observation or referral to a pediatric urologist. We are unable to comment on the appropriateness of referrals for surgical evaluation, the nature of the tests that should be done during the pre-surgical assessment of hydronephrosis, or technical aspects of corrective surgical procedures. Our aim is to focus on the gatekeeper role of general pediatricians and pediatric nephrologists in the care of children with hydronephrosis. The outcomes of those managed by urologists would be the subject of an independent study.

## Conclusions

The results of this single center, retrospective study suggest that prolonged monitoring is suitable for the majority of children with neonatal hydronephrosis and presumed UPJO because there is spontaneous resolution of the lesion. Children with UPJO hydronephrosis that is not severe and in whom there are no questions about renal function can be followed for at least 2 years to allow for spontaneous resolution before referral to urology for radionuclide scans and possible pyeloplasty. In the absence of indications for prompt surgical intervention or worsening hydronephrosis, serial renal sonography can be performed every 6 months. Risk factors for persistence of hydronephrosis are presence at birth and severity at the time of diagnosis. Our study provides an approach for primary care physicians and pediatric nephrologists to manage hydronephrosis by allowing a longer observation period for spontaneous resolution and increased time between follow-up ultrasounds.

## Figures and Tables

**Table 1:** UPJO: patient data.

**Factor**	Number (data available)^*^	**%**
Gender (M:F)	96:40 (136)	71:29
Premature	15 (117)	13
UTI	23 (136)	17
Time of diagnosis (At birth:Post-birth)	87:28 (115)	76:24
Prenatal diagnosis	92 (136)	68
Perinatal complications	53 (130)	41
Age at diagnosis (months)	3.3 ± 9.7	–
Normal VCUG	112 (112)	100

* The numbers in parentheses indicate the number of charts reviewed in which the specific information was available or the number of patients in whom the indicated test was done.

**Table 2:** UPJO: radiology data.

	Number of patients^*^	**Percent of patients**
Unilateral disease	98/136	72
Bilateral disease	38/136	28
Severity of UPJO (for each renal unit):	Number of kidneys	Percent of kidneys
– Mild	105	60
– Mild-to-moderate	30	17
– Moderate	24	14
– Moderate-to-severe	6	3
– Severe	9	5

* The denominator indicates the number of patients in each category with available data.

**Table 3:** UPJO: long-term outcome based on severity of hydronephrosis.

**Outcome**	**≤Mild-to-moderate**	**≥Moderate**
	N = 98^*^	N = 38^*^
Resolved	67	10
Referred to urology	10	20
Improving	1	4
Worsening	1	0
Stable	4	2
Lost to follow-up	15	2

* The numbers refer to patients not renal units.

**Table 4:** UPJO: clinical and radiological risk factors for persistence.

**Factor**	**Frequency of persistence**				P value^*^
		**%**		**%**	
Gender	Male (36/93)	38.7	Female (18/37)	48.6	0.33
Prematurity	Premature (4/13)	30.1	Full term (40/99)	40.4	0.56
UTI	Yes (6/20)	30	No (47/110)	42.7	0.33
Laterality	Unilateral (39/94)	41.5	Bilateral (15/36)	41.7	1
Prenatal US +	Prenatal (39/89)	43.8	Postnatal (14/41)	34.1	0.34
Time of diagnosis	Birth (34/87)	39.1	Later (4/28)	14.3	0.02
Perinatal complications	Complications (23/48)	47.9	No complications (30/82)	36.6	0.22
Severity	≤mild/mod (16/83)	19.3	≥mod (26/36)	72.2	0.0001

* Results represent the findings from the paired analyses.

**Table 5:** UPJO: predictors for persistent hydronephrosis.

**Factor**	**Odds ratio (OR)**	**Confidence interval (CI)**	P value*
Gender	0.67	0.32–1.42	0.3
Prematurity	0.86	0.21–3.55	0.83
UTI	0.55	0.19–1.58	0.27
Laterality	0.76	0.39–1.51	0.44
Time of diagnosis	0.42	0.17–1.05	0.06
Severity	7.71	3.29–18.07	<0.001

* Results represent the findings from the simple logistic regression.

## Abbreviations

APD: anteroposterior pelvic diameter;
SFU: Society for Fetal Urology;
UPJO: ureteropelvic junction obstruction;
UTI: urinary tract infection;
VCUG: voiding cystourethrogram.
